# Disability and Employment Inclusion in a Transforming Economy: Epidemiological Evidence from National Survey Data

**DOI:** 10.1177/00469580261441064

**Published:** 2026-04-13

**Authors:** Abdullah Alduais, Ahmed Alduais

**Affiliations:** 1 Special Educational Needs Unit, English Language Institute, King Abdulaziz University, Jeddah, Saudi Arabia; 2 Norwegian University of Science and Technology, Trondheim, Norway

**Keywords:** disability inclusion, employment, gender inequality, Saudi Arabia, epidemiology, social policy, Vision 2030

## Abstract

Employment inclusion for persons with disabilities is a critical public-health and social-justice priority. In Saudi Arabia, the intersection of disability, gender, and national reform under Vision 2030 presents unique challenges and opportunities for equitable labour participation. This study examined employment patterns, structural determinants, and potential policy pathways to enhance inclusion among Saudis with disabilities. A cross-sectional analytical design was applied using nationally representative data from the 2017 Saudi Disability Survey (n = 1 312 261 working-age individuals). Employment status served as the dependent variable, while severity, difficulty scope, sex, marital status, and access to Ministry of Labor and Social Development (MLSD) services were predictors. Weighted descriptive statistics and grouped logistic regression were used to identify correlates of employment, followed by counterfactual simulations estimating potential gains under alternative policy scenarios. National employment among persons with disabilities was 18.5%, with pronounced disparities by gender and severity. Men were 2.6 times more likely than women to be employed (AOR = 2.64, *P* < .001). Each severity level reduced employment odds by ≈40%, while MLSD service access modestly improved inclusion (AOR = 1.21). Married individuals showed higher participation (AOR = 1.32). Simulations projected that integrated policies combining expanded service coverage and gender-targeted incentives could raise employment inclusion by ≈5% points. Employment inclusion in Saudi Arabia remains strongly shaped by gender and functional severity, with institutional services offering limited but positive effects. Findings highlight the need for multi-sectoral strategies—linking rehabilitation, workplace accessibility, and gender-equity reforms—to advance national inclusion goals. The 2017 survey provides a baseline for evaluating Vision 2030 reforms and future longitudinal assessments of disability employment.

## Introduction

### Background and Rationale

Employment inclusion for persons with disabilities is framed internationally as a matter of human rights and social justice. The Convention on the Rights of Persons with Disabilities affirms the right to work on an equal basis with others and calls for accessible environments and non-discriminatory labour practices. Empirical and policy analyses show that realizing this right requires attention to both workplace adjustments and wider social supports that counter exclusionary norms and practices.^1,2^ Studying employment inclusion is therefore essential for identifying and removing barriers that kerb participation and dignity at work.

A robust theoretical basis for this work comes from the International Classification of Functioning, Disability and Health. The ICF conceptualizes disability as an interaction between health conditions and environmental factors that either enable or restrict participation. This perspective directs attention to modifiable features of places, programs, and organizations that shape access to jobs and career progression.^3-6^ Evidence indicates that barriers span physical design, transport, organizational routines, and supervisory attitudes, all of which influence hiring and retention for people with different impairment profiles.^4,7^

The ICF provides a structure that links health conditions to functioning by grouping concepts into body functions and structures, activities, participation, and environmental factors. In this study, severity level and difficulty scope reflect the body functions and structures domain because they capture the degree to which impairments limit functioning.^8^ Employment status represents the activities and participation domain since it reflects engagement in a socially and economically valued role. Several variables serve as environmental factors, including access to MLSD services, regional variation, and gendered social expectations documented in national policy literature. These elements influence opportunities, supports, and constraints surrounding participation. Aligning the study variables with these ICF domains clarifies how employment inclusion emerges from the interaction between individual functioning and surrounding social and institutional conditions.

The stakes are socioeconomic as well as normative. People with disabilities face higher risks of poverty and exclusion, and gaps in labour-force participation persist across settings. Inclusion in work supports income, social ties, and self-determination, which is why employment is frequently highlighted as a priority outcome in disability policy and practice.^9,10^ Global agendas reinforce this focus. Commitments in the Sustainable Development Goals link disability inclusion to poverty reduction, decent work, and reduced inequalities, and they emphasize the need for policy design grounded in reliable data about barriers and facilitators of participation.^2,9^ In parallel, population-based studies show that credible evidence on prevalence and determinants is crucial for tailoring interventions and monitoring progress.^5,11^ Taken together, these frameworks and findings justify an epidemiological approach to employment inclusion that maps patterns of disadvantage, identifies changeable conditions, and informs practical strategies aligned with international standards.

### National Context: Policy Reforms, Vision 2030, and Gender Inclusion in Saudi Arabia

In Saudi Arabia, disability inclusion and gender equity have become key elements of national transformation under Vision 2030, which emphasizes economic diversification, human capital development, and social modernization. Recent national statistics show rapid changes in female participation in the workforce—rising to over 20%, with women representing one of the fastest-growing segments of the labour market among G20 nations.^12,13^ Despite these advances, gender disparities remain evident across sectors. For instance, women constitute nearly two-thirds of nurses but remain underrepresented in other medical and managerial roles.^14^ This uneven distribution underscores the broader challenge of translating legal reforms into equitable labour outcomes.

Vision 2030 serves as the strategic blueprint for these reforms, aiming to increase female labour-force participation, foster inclusion, and build a sustainable knowledge economy.^12,15^ The program’s social transformation agenda has been supported by new legislation banning workplace gender discrimination, guaranteeing equal pay, and dismantling elements of the guardianship system that previously restricted women’s mobility and employment opportunities.^16,17^ Initiatives such as Gurrah and Wusool provide logistical and financial support for working women, while labour law reforms extend maternity benefits and encourage flexible employment arrangements.^17^ Together, these policies reflect a systematic effort to promote equality, enhance productivity, and align Saudi labour regulations with global standards.

Labour-market transformation is central to the Vision 2030 framework. Economic diversification initiatives—including the Nitaqat program—have expanded opportunities in private and service sectors and promoted greater inclusion of Saudi citizens, especially women.^12^ The rise of female leadership is increasingly visible, with women occupying executive roles in healthcare, education, and urban governance.^14,17^ However, representation in technical fields such as urban planning remains limited, even though women constitute nearly half of the urban population and urbanization is projected to reach 98% by 2030.^15^

Social protection measures also support these structural changes. Vision 2030 programs emphasize civic engagement, education, and skills development to prepare women for future-oriented employment sectors and leadership roles.^18^ Broader efforts to improve social determinants of health and wellbeing—such as equitable access to healthcare, job security, and education—are recognized as essential for sustaining an inclusive workforce.^19^ Saudi Arabia’s gender policies align with international commitments under Sustainable Development Goal 5 (Gender Equality) and the New Urban Agenda, reflecting a growing commitment to global standards while acknowledging ongoing challenges in full implementation.^15^

In this context, understanding disability and employment inclusion acquires particular relevance. The same economic and policy reforms that expanded women’s opportunities are also reshaping labour dynamics for other underrepresented groups, including people with disabilities. As Saudi Arabia advances towards Vision 2030 goals, evidence-based analyses of employment, gender, and disability inclusion are essential for ensuring that modernization benefits all citizens equitably and sustainably.^16,20^

Several national measures relevant to disability inclusion in employment were introduced after the 2017 survey year. These included the establishment of the Authority of People with Disability in 2018,^21^ the amendment to Article 3 of the Labour Law in 2019 to prohibit discrimination in employment,^22^ the National Policy for Promoting Equal Opportunities and Equal Treatment in Employment and Profession approved in 2023,^23^ and the Law on the Rights of Persons with Disabilities enacted in 2023.^24^ Recent analyses also highlighted the role of the 2023 law in advancing disability rights and aligning national policy with international standards.^25^

### Empirical Gap in National Research on Disability Employment

Despite progress in disability and labor inclusion policies, national research remains fragmented and limited in scope. Studies often rely on localized or sector-specific data, preventing a coherent epidemiological understanding of disability and employment at the national level. In many countries, major surveys are underutilized, and data integration across sources remains incomplete, resulting in partial and sometimes inconsistent representations of employment disparities.^26,27^ Research also tends to prioritize formal employment, neglecting informal or precarious work where many individuals with disabilities are active.^28^ Moreover, analyses usually focus on employment rates rather than multidimensional employment quality, overlooking critical welfare and psychosocial outcomes.^29,30^

Conceptually, most studies adopt a narrow view of disability that fails to capture heterogeneity by gender, ethnicity, or social background.^31^ Underreporting caused by non-disclosure of hidden disabilities further distorts national prevalence estimates.^32^ Methodologically, few investigations distinguish between selection and causal effects of disability on employment, limiting understanding of mechanisms behind observed disparities.^33^ Collectively, these limitations highlight a need for nationally representative, population-based analyses using validated indicators. The present study addresses this gap by applying an epidemiological framework to examine disability employment inclusion across Saudi Arabia, using data from the 2017 national survey to provide a comprehensive, policy-relevant evidence base.

### Purpose of the Present Study

Published national studies using the 2017 GASTAT disability survey have focussed on specific functional domains rather than employment outcomes. Recent work reported national estimates of mobility difficulty, communication difficulty, and related sociodemographic patterns, but did not assess labour-market participation or structural determinants of employment.^34,35^ Analyses of broader health and mental health indicators have also used national survey data without addressing employment inclusion.^36^ No published studies have used the 2017 disability microdata to examine employment disparities, and the 2023 disability release provides only aggregate percentages without accessible microdata for modelling. These limitations confirmed the need for the present study, which offered adjusted estimates of employment inclusion and examined structural correlates using a national epidemiological approach.

This study has 4 aims: (1) To examine the epidemiological distribution of employment and labour-force participation among Saudis with disabilities by sex, severity, and difficulty scope; (2) To identify the sociodemographic and policy factors (marital status, service access) associated with employment inclusion; (3) To estimate adjusted associations between disability characteristics and employment outcomes using grouped logistic regression; and (4) To project potential employment gains under alternative inclusion policy scenarios based on national survey evidence. For the inferential analysis purposes, we also considered these hypotheses: H1: Employment participation among persons with disabilities varies significantly by sex and severity of disability; H2: Access to Ministry of Labor and Social Development (MLSD) services increases the likelihood of being employed; H3: Marital status contributes to employment differences, reflecting social-structural influences in the Saudi context; and H4: Policy simulation models will show measurable employment gains under expanded service and education scenarios. Thus, this study focuses on 4 analytical dimensions—labour-market, policy-support, social-structural, and demographic/functional baseline—representing distinct but interrelated aspects of disability inclusion.

## Methods

### Study Design

This study adopted a cross-sectional analytical design based on nationally representative data from the Saudi Disability Survey 2017, conducted by the GASTAT. The research followed the Strengthening the Reporting of Observational Studies in Epidemiology (STROBE) guidelines for cross-sectional studies, ensuring transparent reporting of design, variables, and analytical procedures.^37^ The study used secondary data collected through structured household surveys designed to assess the demographic, functional, and socioeconomic characteristics of individuals with disabilities across the Kingdom of Saudi Arabia. A cross-sectional design was appropriate given the primary aim of estimating the prevalence and correlates of employment inclusion and other social indicators among persons with disabilities at a single point in time. Although causal inference is limited in such designs, the national coverage and stratified sample provide robust population-level estimates. The design allows epidemiological profiling of disability within its demographic and policy context, consistent with international practices for population-based disability research.^38^

### Setting

The study was conducted in the Kingdom of Saudi Arabia, encompassing all 13 administrative regions as defined by the General Authority for Statistics (GASTAT). Data were drawn from the 2017 Saudi Disability Survey, implemented as part of the Population Characteristics Survey. Fieldwork was carried out between April and May 2017, covering 33 575 sampled households across both urban and rural areas. The survey’s design supported regional disaggregation, allowing estimation of disability prevalence and employment inclusion by sex, age, and severity level. Data collection was completed using GASTAT’s standardized Computer-Assisted Personal Interviewing (CAPI) system to maintain reliability across field teams and geographic zones. Enumerators collected information on demographic characteristics, health status, and functional limitations based on the International Classification of Functioning, Disability and Health (ICF) framework.^8^ The inclusion of all administrative regions from the major metropolitan areas of Riyadh and Makkah to smaller provinces such as Najran and Al-Baha provided a robust foundation for assessing both national and regional disability patterns. This comprehensive geographic coverage enhances the external validity of findings and situates disability within Saudi Arabia’s public-health and socio-economic development contexts.

### Participants

Participants in this study were individuals identified within the 33 575 households included in the Saudi Disability Survey 2017, conducted by the GASTAT. The survey used a stratified, multistage probability sampling design to ensure national representativeness of Saudi citizens across the country’s 13 administrative regions. Each sampling stratum combined regional and urban–rural classifications to achieve proportional inclusion of population clusters. All usual household members were eligible for enumeration, and individuals were classified as having a disability if they reported “some difficulty,” “a lot of difficulty,” or “cannot do at all” in at least 1 of 6 functional domains: vision, hearing, mobility, cognition, self-care, and communication. These domains were based on the Washington Group Short Set on Functioning, which has been internationally validated for censuses and national surveys.^38,39^ The analytic dataset for this study included all persons aged 15 years and older with a reported difficulty in at least 1 domain, totalling 2 429 521 individuals. Children aged under 15 years (n = 133 462) were retained for prevalence estimation in Table 8 but excluded from all employment and marital-status analyses because these outcomes were not applicable to this age group. Weighting adjustments provided by GASTAT were applied to ensure alignment between the sample and national population estimates for 2017.

### Variables

This study analysed variables derived from the 2017 Saudi Disability Survey to examine the determinants of employment inclusion among persons with disabilities. Following STROBE guidelines,^37^ variables were classified into outcome, exposure, and contextual domains consistent with the study’s conceptual and analytical frameworks. The primary outcome variable was employment status, coded dichotomously as employed (1) or not employed (0). Employment included individuals reporting paid work, self-employment, or work without pay in family businesses, while those classified as unemployed, retired, studying, or homemakers were grouped as not employed.

The main exposure variables were disability severity and difficulty scope. Severity was measured at 3 levels (mild, severe, and extreme) based on self-assessed functional limitations. Difficulty scope distinguished between single and multiple difficulties, reflecting the cumulative burden of impairment. Contextual predictors included sex, age group, marital status, and MLSD service access (receipt of at least 1 service from the Ministry of Labor and Social Development). Regional location was included to capture spatial heterogeneity in inclusion outcomes, while population baseline characteristics from the 2017 Population Characteristics Survey were used for denominator-based prevalence estimates.

The operationalization of all variables is summarized in Table 1, which links each dataset sheet to its conceptual dimension: labour-market, policy-support, social-structural, and demographic/functional baseline. This structure ensured coherence between analytical variables and the broader theoretical framework of disability inclusion in Saudi Arabia. Variables were defined consistently with international disability measurement standards, including the International Classification of Functioning, Disability and Health^8^ and the Washington Group Short Set on Functioning.^39^ It should be noted that all variables used in this analysis were categorical, and their operational definitions, classifications, and transformations were described in this section instead of 2 sections as recommended in the STROBE checklist (items 7 and 11). For the logistic regression models, the dependent variable “employed” was coded as 1 for respondents reporting paid work, self-employment, or unpaid work in a family business, and 0 for all other categories (including retirement, homemaking, student status, unemployment, and inability to work).

**Table 1.** Dataset Structure.

Sheet no.	Dataset title	Population scope/ source	Key variables included	Dimension	Analytical role
1-9	Employment Status (All, Single, Multiple; by sex)	Disability Survey 2017	Employment status, severity, sex, difficulty scope	Labour-market dimension	Core dependent variable (employment inclusion)
10	Services Provided by the Ministry of Labor and Social Development	Disability Survey 2017	Services received (physical, in-kind, home care, day care, accommodation, other, total), sex	Policy-support dimension	Indicator of institutional inclusion support
11-13	Marital Status (All, Single, Multiple; by sex)	Disability Survey 2017	Marital status (never married, married, divorced, widowed), Sex	Social-structural dimension	Sociodemographic context (family & gender norms)
14	Population by Sex, Administrative Area and Nationality	Population Characteristics Survey 2017	Sex, region (13 administrative areas), nationality (Saudi/Non-Saudi)	Demographic baseline dimension	Provides regional and citizenship context for national comparison
15	Population by Sex, Age Group and Nationality	Population Characteristics Survey 2017	Sex, age group (standard bands), Nationality (Saudi/Non-Saudi)	Demographic baseline dimension	Age-sex distribution for comparison with disabled population
16	Saudi Population by Sex, Administrative Area and Difficulty Status	Population Characteristics Survey 2017	Sex, region, difficulty status (with/without difficulty)	Functional baseline dimension	Shows spatial distribution of disability occurrence
17	Saudi Population by Age Group, Sex and Difficulty Status	Population Characteristics Survey 2017	Age group, sex, difficulty status (with/without difficulty)	Functional baseline dimension	Establishes prevalence of disability by age and sex for contextualization

### Data Sources and Measurement

The Saudi Disability Survey 2017, conducted by the General Authority for Statistics (GASTAT), provides the most comprehensive and nationally representative dataset on disability to date. It covered 33 575 households across all administrative regions using the 2010 census frame and Computer-Assisted Personal Interviewing to ensure data reliability and coverage.^40^ The survey captures detailed information on type and severity of difficulty—mobility (29%), vision (24%), and communication (10%)—and distinguishes mild (4.1%) from severe or extreme (2.9%) forms.^41^ Unlike smaller or clinical studies, it also records demographic, geographic, and functional profiles, making it suitable for policy-relevant epidemiological analysis. Its indicators align with international disability frameworks, and the database has already supported population-based analyses of cognitive, mobility, hearing, and communication difficulties.^42,43^ A cross-sectional epidemiological design is appropriate for this dataset because it enables estimation of prevalence and patterning of disability at a specific time point, allowing comparison across demographic and regional subgroups. This approach efficiently identifies associations between disability, employment, and social determinants in a large national population, providing foundational evidence for longitudinal or interventional research in the future.^40,41^

All variables were drawn from the Saudi Disability Survey 2017, conducted by the GASTAT as part of the national Population Characteristics Survey. The dataset comprises 17 linked sheets organized across 4 conceptual dimensions: (1) labour-market indicators, (2) policy-support indicators, (3) social-structural indicators, and (4) demographic and functional baselines (see Table 2). Sheets 1 to 13 were derived from the Disability Survey, while Sheets 14 to 17 were drawn from the Population Characteristics Survey, ensuring comprehensive coverage of both disability-specific and general population data.

**Table 2.** Analysis Plan.

Stage	Analytical focus	Variables/datasets (sheets)	Main analyses/techniques	Expected outputs	Planned presentation (minimum)
1. Data Preparation	Integrate employment, service, and marital data; harmonize coding	Sheets 1-13 (Disability Survey 2017) + Sheets 14-17 (Population Characteristics Survey 2017)	Data merging, recoding, creation of binary outcomes (Employment = 1/0; LFP = 1/0)	Clean analytic file with consistent variable labels	—
2. Descriptive Statistics	Summarize employment and labour-force participation among persons with disabilities	Sheets 1-9	Frequencies and percentages by sex, severity, and difficulty scope	Employment distribution by key predictors	Tables 3-5: Employment status by sex × severity × scope Figure 1: Bar chart of employment rate by severity and sex
3. Services Access	Describe MLSD service provision as indicator of institutional inclusion	Sheet 10	Frequency distribution, % by service type and sex	Prevalence of each service type	Table 6: MLSD services by type and sex
4. Marital Status Patterns	Examine family and gender structure among persons with disabilities	Sheets 11-13	Cross-tabulations and % by sex × marital status	Marital status distribution across difficulty scopes	Table 7: Marital status by sex and scope
5. Baseline Population Context	Compare disabled vs general population profiles	Sheets 14-17	% Distribution by age, sex, nationality, and region; prevalence of disability	National baseline proportions	Table 8: Population and disability prevalence by age × sex × region
6. Regression Models	Identify determinants of employment inclusion	Merged dataset from Sheets 1-13 + services indicator	Grouped logistic regression (logit link); report adjusted odds ratios (AORs) + 95% CI	Predictors of employment	Table 9: Logistic regression results for employment inclusion
7. Interaction and Policy Effects	Test moderating role of services and gender	Employment × MLSD services × severity	Interaction analysis within logistic model	Marginal effects and significance of service access	Figure 2: Predicted probabilities of employment by severity × service access
8. Comparative Interpretation	Position disability results within national context	Merge baseline (Sheets 14-17) with model predictions	Comparison of employment gaps between disabled and non-disabled	Summary indicator of inclusion gap	Figure 2: Comparative employment rates for disabled vs total population
9. Scenario Simulations	Estimate impact of hypothetical policy changes	Regression coefficients from Stage 6	Counterfactual simulation (e.g., +10% service coverage or +10% tertiary education)	Estimated employment gains	Table 10: Predicted employment gains under policy scenarios

The labour-market dimension included indicators of employment status, severity, and difficulty scope, which formed the basis for Tables 3 to 5 in the Results section. The policy-support dimension captured services provided by the MLSD, as recorded in Sheet 10, while the social-structural dimension contained marital status and household-level characteristics (Sheets 11-13). Demographic and functional baseline data from Sheets 14 to 17 provided contextual information on population distribution by age, sex, and region, enabling prevalence estimates and normalization of employment indicators.

**Table 3.** Employment Status of Saudis with Disabilities by Severity, Sex and Difficulty Scope (All Difficulties Combined) Based on the Disability Survey 2017, General Authority for Statistics.

Employment category	Mild (n, %)	Severe (n, %)	Extreme (n, %)	Total (n, %)
Paid work	171 980 (13.1) [*F* 21 852 (1.7)]	51 949 (4.0) [*F* 8050 (0.6)]	9632 (0.7) [*F* 1431 (0.1)]	233 561 (17.8) [*F* 31 333 (2.4)]
Work without pay	2856 (0.2) [*F* 319 (0.0)]	1921 (0.1) [*F* 0 (0.0)]	648 (0.0) [*F* 395 (0.0)]	5425 (0.4) [*F* 714 (0.1)]
Employer employs	9613 (0.7) [*F* 828 (0.1)]	2148 (0.2) [*F* 0 (0.0)]	724 (0.1) [*F* 0 (0.0)]	12 485 (1.0) [*F* 828 (0.1)]
Employer not hired	12 365 (0.9) [*F* 0 (0.0)]	3630 (0.3) [*F* 0 (0.0)]	347 (0.0) [*F* 0 (0.0)]	16 342 (1.2) [*F* 0 (0.0)]
Unemployed – ever worked	18 586 (1.4) [*F* 1662 (0.1)]	7998 (0.6) [*F* 0 (0.0)]	2526 (0.2) [*F* 0 (0.0)]	29 110 (2.2) [*F* 1662 (0.1)]
Unemployed – never worked	37 608 (2.9) [*F* 13 632 (1.0)]	25 048 (1.9) [*F* 8785 (0.7)]	8707 (0.7) [*F* 3622 (0.3)]	71 363 (5.4) [*F* 26 039 (2.0)]
Student	46 543 (3.5) [*F* 22 782 (1.7)]	26 979 (2.1) [*F* 15 289 (1.2)]	6773 (0.5) [*F* 2045 (0.2)]	80 295 (6.1) [*F* 40 116 (3.1)]
Housewife	240 378 (18.3) [*F* 240 378 (18.3)]	114 658 (8.7) [*F* 114 658 (8.7)]	26 239 (2.0) [*F* 26 239 (2.0)]	381 275 (29.1) [*F* 381 275 (29.1)]
Retired	176 764 (13.5) [*F* 9819 (0.7)]	71 938 (5.5) [*F* 5294 (0.4)]	18 909 (1.4) [*F* 676 (0.1)]	267 611 (20.4) [*F* 15 789 (1.2)]
Other	66 788 (5.1) [*F* 42 121 (3.2)]	85 936 (6.6) [*F* 54 677 (4.2)]	62 070 (4.7) [*F* 37 267 (2.8)]	214 794 (16.4) [*F* 134 065 (10.2)]
Total	783 481 (59.7) [*F* 353 393 (26.9)]	392 205 (29.9) [*F* 206 753 (15.8)]	136 575 (10.4) [*F* 71 675 (5.5)]	1 312 261 (100.0) [*F* 631 821 (48.2)]

*Note.* Bracketed values represent females (F); unbracketed totals refer to both sexes combined. Percentages are calculated relative to the grand total (N = 1 312 261).

**Table 4.** Single Difficulty Only.

Employment category	Mild (n, %)	Severe (n, %)	Extreme (n, %)	Total (n, %)
Paid work	140 780 (19.4) [*F* 18 411 (2.5)]	38 498 (5.3) [*F* 7000 (1.0)]	7139 (1.0) [*F* 1431 (0.2)]	186 417 (25.6) [*F* 26 842 (3.7)]
Work without pay	1125 (0.2) [*F* 319 (0.0)]	1192 (0.2) [*F* 0 (0.0)]	648 (0.1) [*F* 395 (0.1)]	2965 (0.4) [*F* 714 (0.1)]
Employer employs	7542 (1.0) [*F* 828 (0.1)]	1182 (0.2) [*F* 0 (0.0)]	397 (0.1) [*F* 0 (0.0)]	9121 (1.3) [*F* 828 (0.1)]
Employer not hired	8168 (1.1) [*F* 0 (0.0)]	1784 (0.2) [*F* 0 (0.0)]	302 (0.0) [*F* 0 (0.0)]	10 254 (1.4) [*F* 0 (0.0)]
Unemployed – ever worked	13 125 (1.8) [*F* 690 (0.1)]	4444 (0.6) [*F* 0 (0.0)]	166 (0.0) [*F* 0 (0.0)]	17 735 (2.4) [*F* 690 (0.1)]
Unemployed – never worked	26 298 (3.6) [*F* 10 715 (1.5)]	11 942 (1.6) [*F* 4839 (0.7)]	2586 (0.4) [*F* 1286 (0.2)]	40 826 (5.6) [*F* 16 840 (2.3)]
Student	40 174 (5.5) [*F* 19 912 (2.7)]	22 640 (3.1) [*F* 13 411 (1.8)]	3992 (0.5) [*F* 1496 (0.2)]	66 806 (9.2) [*F* 34 819 (4.8)]
Housewife	152 968 (21.0) [*F* 152 968 (21.0)]	52 643 (7.2) [*F* 52 643 (7.2)]	10 455 (1.4) [*F* 10 455 (1.4)]	216 066 (29.7) [*F* 216 066 (29.7)]
Retired	99 421 (13.7) [*F* 6672 (0.9)]	29 123 (4.0) [*F* 2814 (0.4)]	4097 (0.6) [*F* 0 (0.0)]	132 641 (18.2) [*F* 9486 (1.3)]
Other	27 374 (3.8) [*F* 17 798 (2.4)]	12 213 (1.7) [*F* 8960 (1.2)]	4455 (0.6) [*F* 2754 (0.4)]	44 042 (6.1) [*F* 29 512 (4.1)]
Total	516 975 (71.1) [*F* 228 313 (31.4)]	175 661 (24.2) [*F* 89 667 (12.3)]	34 237 (4.7) [*F* 17 817 (2.5)]	726 873 (100.0) [*F* 335 797 (46.2)]

*Note.* Bracketed values represent females (F); unbracketed totals refer to both sexes combined. Percentages use the single-difficulty grand total N = 726 873.

**Table 5.** Multiple Difficulties Only.

Employment category	Mild (n, %)	Severe (n, %)	Extreme (n, %)	Total (n, %)
Paid work	31 200 (5.3) [*F* 3441 (0.6)]	13 451 (2.3) [*F* 1050 (0.2)]	2493 (0.4) [*F* 0 (0.0)]	47 144 (8.1) [*F* 4491 (0.8)]
Work without pay	1731 (0.3) [*F* 0 (0.0)]	729 (0.1) [*F* 0 (0.0)]	0 (0.0) [*F* 0 (0.0)]	2460 (0.4) [*F* 0 (0.0)]
Employer employs	2071 (0.4) [*F* 0 (0.0)]	966 (0.2) [*F* 0 (0.0)]	327 (0.1) [*F* 0 (0.0)]	3364 (0.6) [*F* 0 (0.0)]
Employer not hired	4197 (0.7) [*F* 0 (0.0)]	1846 (0.3) [*F* 0 (0.0)]	45 (0.0) [*F* 0 (0.0)]	6088 (1.0) [*F* 0 (0.0)]
Unemployed – ever worked	5461 (0.9) [*F* 972 (0.2)]	3554 (0.6) [*F* 0 (0.0)]	2360 (0.4) [*F* 0 (0.0)]	11 375 (1.9) [*F* 972 (0.2)]
Unemployed – never worked	11 310 (1.9) [*F* 2917 (0.5)]	13 106 (2.2) [*F* 3946 (0.7)]	6121 (1.0) [*F* 2336 (0.4)]	30 537 (5.2) [*F* 9199 (1.6)]
Student	6369 (1.1) [*F* 2870 (0.5)]	4339 (0.7) [*F* 1878 (0.3)]	2781 (0.5) [*F* 549 (0.1)]	13 489 (2.3) [*F* 5297 (0.9)]
Housewife	87 410 (14.9) [*F* 87 410 (14.9)]	62 015 (10.6) [*F* 62 015 (10.6)]	15 784 (2.7) [*F* 15 784 (2.7)]	165 209 (28.2) [*F* 165 209 (28.2)]
Retired	77 343 (13.2) [*F* 3147 (0.5)]	42 815 (7.3) [*F* 2480 (0.4)]	14 812 (2.5) [*F* 676 (0.1)]	134 970 (23.1) [*F* 6303 (1.1)]
Other	39 414 (6.7) [*F* 24 323 (4.2)]	73 723 (12.6) [*F* 45 717 (7.8)]	57 615 (9.8) [*F* 34 513 (5.9)]	170 752 (29.2) [*F* 104 553 (17.9)]
Total	266 506 (45.5) [*F* 125 080 (21.4)]	216 544 (37.0) [*F* 117 086 (20.0)]	102 338 (17.5) [*F* 53 858 (9.2)]	585 388 (100.0) [*F* 296 024 (50.6)]

*Note.* Bracketed values represent females (F); unbracketed totals refer to both sexes combined. Percentages are calculated relative to N = 585 388 (multiple-difficulty population).

All survey instruments were administered in Arabic by trained GASTAT enumerators using standardized questionnaires aligned with the ICF.^8^ Functional difficulty was measured using the Washington Group Short Set on Functioning (WG-SS), which defines disability through self-reported limitations in core domains of activity and participation.^38,39^ Data underwent double-entry verification and consistency checks by GASTAT before public release. Variables were cleaned, recoded, and integrated into a unified analytical file preserving all weighting coefficients to maintain national representativeness.

### Bias

To minimize bias in data collection and analysis, several methodological safeguards were applied by the GASTAT during the implementation of the 2017 Disability Survey. According to the official methodology,^44^ all enumerators underwent centralized training on disability concepts and the administration of the Washington Group Short Set on Functioning, with emphasis on accurate translation and culturally appropriate interviewing. Supervisors conducted daily field audits to ensure adherence to standardized procedures, and completed questionnaires were verified electronically using GASTAT’s CAPI system, which automatically flagged missing or inconsistent responses. A multistage quality-control process further included random re-visits to households and centralized data consistency checks before tabulation.

Potential sources of bias inherent to household surveys—such as self-report bias, coverage bias, and non-differential misclassification—were mitigated through methodological design. The use of standardized response categories (no difficulty, some difficulty, a lot of difficulty, cannot do at all) reduced subjective interpretation, while inclusion of both urban and rural clusters improved representativeness across socio-economic strata. Analytical bias was reduced by applying sampling weights provided by GASTAT to align estimates with the official 2017 population projections. Although self-reported functional limitations may underestimate mild impairments, the survey’s design and field controls make it one of the most reliable national sources for disability epidemiology in Saudi Arabia. Missing data on employment status, severity level, MLSD service access, and marital status were minimal (each <1%) and were handled using listwise deletion. Sensitivity checks indicated that estimates were unchanged when models were re-estimated with missing categories retained, suggesting no material bias from this approach.

### Study Size

The study analysed nationally representative data from the Saudi Disability Survey 2017. In total, 1 445 723 Saudis were identified as having at least 1 functional difficulty (all ages). Because employment outcomes are only meaningful for working-age persons, the analytic sample for employment models comprised 1 312 261 individuals aged ≥ 15 years with disability. Sampling followed a stratified multistage probability design, and GASTAT weights were applied to restore national population proportions in estimation.^44^ Large, representative samples are recommended for disability research to secure precision for prevalence, subgroup analyses, and policy-relevant indicators.^45,46^

### Statistical Methods

All analyses were conducted according to the plan summarized in Table 2. Because all study variables were categorical, frequencies and percentages were calculated to describe employment, service access, marital status, and demographic distributions. These descriptive statistics were disaggregated by sex, severity, and difficulty scope to illustrate population-level inclusion patterns. Employment indicators were computed as proportions of the total number of working-age persons with disabilities, using GASTAT-provided weights to ensure representativeness.

For inferential analysis, binary logistic regression models were fitted to identify predictors of employment inclusion among persons with disabilities. Adjusted odds ratios (AORs) and 95% confidence intervals (CIs) were estimated for sex, severity, difficulty scope, marital status, and MLSD service access, controlling for region and age group. The models were used to generate predicted probabilities of employment under varying exposure conditions, following marginal-effects procedures for cross-sectional survey data.^47,48^

Interaction analyses assessed whether MLSD service access moderated the association between severity and employment, and between sex and employment, using multiplicative interaction terms within the logistic models. Finally, counterfactual simulations were conducted to estimate the potential employment gains under hypothetical policy changes—such as increasing MLSD service coverage or female participation—by applying adjusted odds ratios from the regression models to baseline probabilities. These simulated effects were summarized in Table 10 as predicted employment gains under alternative policy scenarios. All analyses followed STROBE guidelines for cross-sectional data.^37^ Statistical computations were performed using IBM SPSS Statistics (Version 29.0), and all figures were produced in R (Version 4.3) using standard plotting libraries.

Individual MLSD service types were not included as separate predictors because several categories had very small cell sizes and substantial overlap, which created collinearity and unstable estimates. Further, the structure of the 2017 Disability Survey dataset does not permit estimation of statistical interaction terms (eg, sex × severity; sex × MLSD service access) because the microdata are not available and the analysis relies on grouped national totals rather than individual-level observations. Grouped logistic regression provides valid marginal associations but cannot compute cross-classified joint distributions required for interaction effects. For transparency, this limitation is acknowledged and addressed further in the Discussion.

The policy simulations were based on standard marginal-effects calculations and assumed linear and additive effects of the predictors. The AORs were treated as constant across intervention scales, and no additional interaction or saturation effects were modelled. The simulations also assumed that the policy environment remains comparable to the conditions represented in the 2017 dataset.

## Results

Table 3, provides a national overview of employment participation among Saudis with disabilities, integrating gender and severity in 1 unified display. Paid work represents the largest single employment category (17.8%), though only 2.4% of all persons with disabilities are females in paid positions. Non-labour-force roles dominate the distribution, led by the housewife category (29.1%) and retired individuals (20.4%), confirming the predominance of domestic and post-employment statuses in the disabled population. Employment opportunities narrow progressively with severity: mild difficulty accounts for nearly 60% of the total population with disabilities, yet only a minority engage in paid or entrepreneurial work. Female representation is particularly constrained across all employment types except for the housewife category, where women constitute the entirety of participants. The “other” classification expands with severity, suggesting diverse forms of exclusion or unclassified activity. Overall, this epidemiological distribution demonstrates how gender and severity jointly shape patterns of economic inactivity and dependency within Saudi Arabia’s disabled population.

Single-difficulty cases are concentrated in the mild category, which accounts for just over 70% of the population (Table 4). Paid work is most common within mild difficulty but declines at higher severity. Female participation in paid employment remains limited in every severity category, while the housewife category accounts for a large segment of the single-difficulty population and all of it is female, consistent with sex-specific coding in the survey. Retirement absorbs a notable share of single-difficulty cases, particularly among those with mild or severe limitations, suggesting early exit from the labour market. Student status is comparatively visible here, indicating that individuals with a single impairment are more likely to remain in education than those with multiple difficulties. Unemployment categories together remain modest but persistent across severities, and the small employer categories illustrate limited entrepreneurial engagement. The pattern indicates that, even without multiple impairments, many individuals with disabilities remain outside paid employment, with distinct gendered distributions across roles.

Among individuals with multiple disabilities, participation in paid employment falls to only 8% of the population, confirming limited labour-force integration in this group (Table 5). Females represent less than one percentage point of all paid positions, while their concentration in the *housewife* category reaches 28%—the largest single category in the table. Retired individuals (23%) and those classified as *other* (29%) together account for more than half the multiple-difficulty population, indicating strong withdrawal from formal or even informal employment. The unemployed categories remain small but stable across severity levels, suggesting that most persons with multiple impairments are not seeking work at all. The student proportion (2%) demonstrates minimal educational engagement, declining further with greater severity. Severity gradients are steeper than in the single-difficulty dataset: mild difficulty comprises nearly half of all multiple-disability cases, but only 5% of this group is employed. The data portray a population characterized by dependency, low educational participation, and near-absence of economic inclusion, especially for females, whose participation outside the home remains almost entirely restricted to unpaid or domestic roles.

Across the 3 datasets—representing all, single, and multiple difficulties—consistent patterns emerge in the distribution of employment and non-employment roles among Saudis with disabilities. Employment rates decline systematically with increasing severity and with the accumulation of multiple functional limitations. In the *all-difficulties* dataset (Table 3), about 18% of individuals report paid work, dropping to 13% in single-difficulty cases (Table 4) and to only 8% among those with multiple disabilities (Table 5). As functional limitation intensifies, the *housewife* and *retired* categories dominate, together comprising roughly half of each population subgroup. Female participation in paid work remains exceptionally limited in every scenario, rarely exceeding 2% of the total, while nearly all females are classified as housewives. The *student* category appears more prominent among those with single difficulties, reflecting continued educational engagement in less severe conditions, but this proportion declines sharply in multiple-difficulty cases. Meanwhile, the *other* classification expands with severity, indicating diverse forms of exclusion and unrecorded economic inactivity. Collectively, these patterns portray a population characterized by structural dependence, early withdrawal from formal employment, and pronounced gender asymmetry—trends that persist regardless of the number or severity of disabilities.

**Figure 1.**
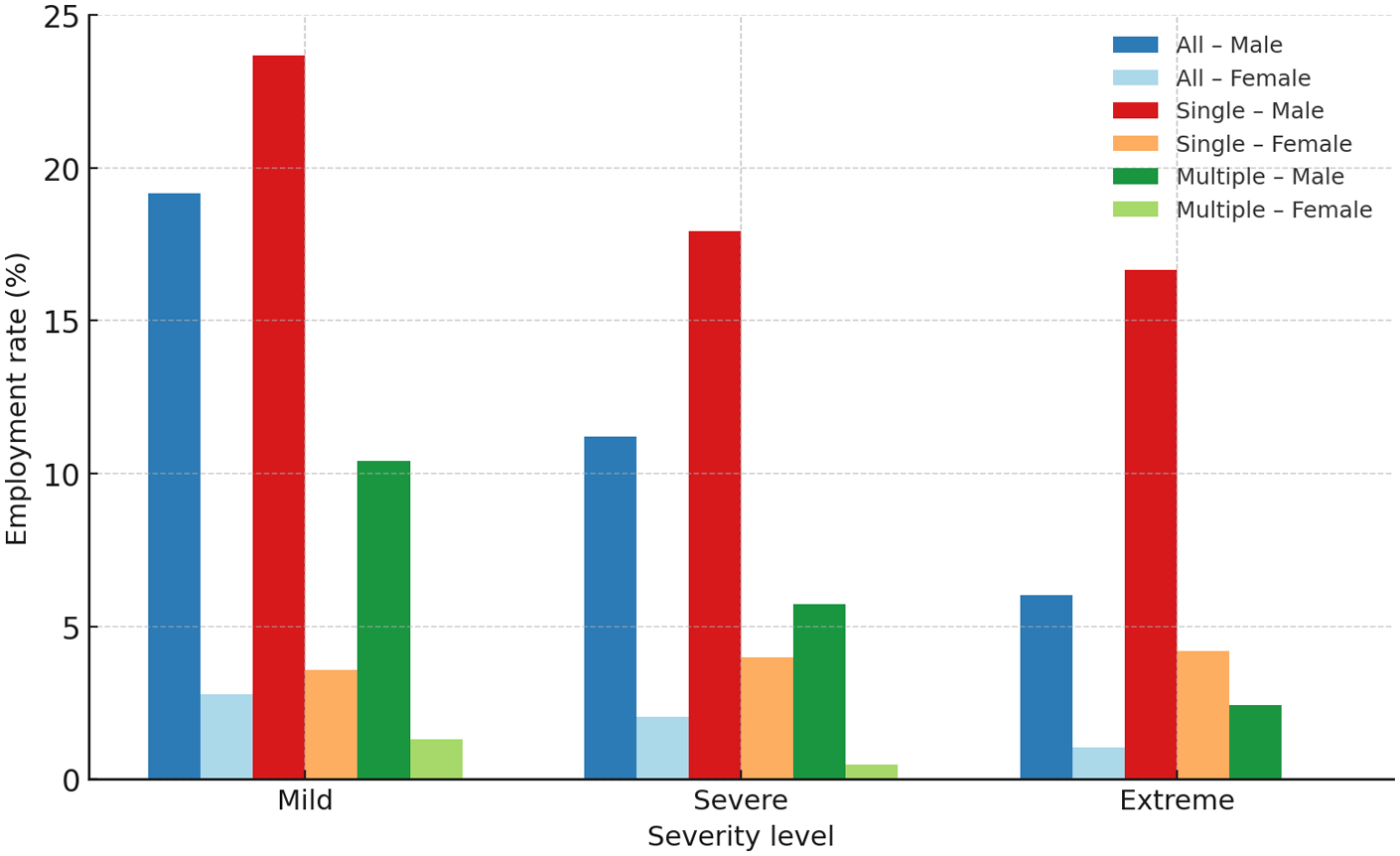
Employment rate (%) among Saudis with disabilities by severity, sex, and difficulty scope (Disability Survey 2017).

The bar chart in Figure 1 visualizes the decline in employment rates with increasing severity across all disability scopes. Males consistently maintain higher employment rates than females, and this gap widens with severity. Among those with a single difficulty, employment participation is highest—particularly for mild difficulty—while individuals with multiple difficulties show markedly lower rates, rarely exceeding 10%. Female employment remains extremely low across all conditions, seldom surpassing 4%. The visual distinction between male and female bars across the 3 scopes reinforces the structural gender divide observed in the tabular data, while also highlighting how the accumulation of multiple impairments further limits labour-force inclusion.

Service access under the MLSD reveals moderate gender variation in both reach and type (Table 6). Women constitute a slightly larger share of total recipients (55%), yet their pattern of service use differs from that of men. Males dominate in physical and in-kind aid, which together account for about 9% of all beneficiaries, reflecting greater male representation in material-support programs. In contrast, females receive proportionally more home and day-care services, indicating alignment with family-care and custodial roles. Accommodation and day-care provision remain limited overall, together representing barely 1% of all beneficiaries, suggesting low institutional-care coverage nationwide. The “other services” category, comprising 3% of recipients, likely captures miscellaneous or region-specific forms of assistance. When combined, the distribution shows that MLSD service provision prioritizes essential and maintenance-based support over rehabilitative or employment-linked services, with a discernible gender tilt towards caregiving assistance for women and physical/material support for men. This pattern highlights both progress in coverage and persisting gender-role differentiation in institutional inclusion.

**Table 6.** MLSD Services Received by Saudis with Disabilities by Type and Sex Based on the Disability Survey 2017, General Authority for Statistics.

Service type	Male (n, %)	Female (n, %)	Total (n, %)
Physical aid	13 043 (3.7)	16 046 (4.5)	29 089 (8.2)
In-kind assistance	18 419 (5.2)	14 600 (4.1)	33 019 (9.3)
Home care	3073 (0.9)	4397 (1.2)	7470 (2.1)
Day care	1354 (0.4)	2188 (0.6)	3542 (1.0)
Accommodation	498 (0.1)	547 (0.2)	1045 (0.3)
Other services	7250 (2.0)	4829 (1.4)	12 079 (3.4)
Total recipients	161 007 (45.5)	192 607 (54.5)	353 614 (100.0)

*Note.* Percentages are calculated relative to N = 353 614 (people receiving at least 1 MLSD service). Several service types had limited sample counts, which prevented reliable stratified estimation of employment outcomes by specific service category.

Marriage is the dominant status in every group, yet its share is much higher among men than women (Table 7). Women show a very large widowed share, especially in the multiple-difficulties group where widowed reaches 42.6%, suggesting the joint influence of age structure and severity on family status. Men are more often never married across scopes, which aligns with the employment patterns seen earlier where men with disabilities face participation constraints that can affect marriage prospects. Divorced status remains small but consistently higher among women than men. Moving from single to multiple difficulties shifts the distribution towards widowhood for both sexes and reduces the married share for women more than for men. These patterns support the social-structural dimension of the study, indicating that gender roles and the accumulation of impairments shape household formation and caregiving arrangements.

**Table 7.** Marital Status of Saudis with Disabilities by Sex and Difficulty Scope Using the Disability Survey 2017, General Authority for Statistics.

Sex × Difficulty scope	Never married (n, %)	Married (n, %)	Divorced (n, %)	Widowed (n, %)	Total (n, %)
All difficulties – Male	129 510 (19.0)	522 057 (76.7)	13 416 (2.0)	15 457 (2.3)	680 440 (100.0)
All difficulties – Female	94 928 (15.0)	317 228 (50.2)	23 688 (3.7)	195 977 (31.0)	631 821 (100.0)
Single difficulty – Male	77 637 (19.9)	302 382 (77.3)	7570 (1.9)	3487 (0.9)	391 076 (100.0)
Single difficulty – Female	56 464 (16.8)	195 631 (58.3)	13 936 (4.2)	69 766 (20.8)	335 797 (100.0)
Multiple difficulties – Male	51 873 (17.9)	219 675 (75.9)	5846 (2.0)	11 970 (4.1)	289 364 (100.0)
Multiple difficulties – Female	38 464 (13.0)	121 597 (41.1)	9752 (3.3)	126 211 (42.6)	296 024 (100.0)

*Note.* Percentages are computed within each row. Totals across rows are not additive because “All difficulties” includes single and multiple difficulties.

National disability prevalence in 2017 stood at 7.1%, slightly higher among males (7.3%) than females (6.9%; Table 8). Age gradients are pronounced: prevalence remains below 5% until the mid-forties but rises rapidly thereafter, exceeding 40% among those aged 75 years and older. This trajectory reflects the cumulative effect of chronic conditions and ageing on functional ability. Regional disparities are evident yet moderate. Southern and peripheral regions—Asir, Hail, and Jazan—register the highest rates (≥8%), while Najran and Qassim show the lowest (<6%). Large metropolitan areas such as Riyadh and Makkah present mid-range levels around 7% to 8%, consistent with younger, more urban populations. Female prevalence exceeds male in a few regions (eg, Makkah and Qassim), likely due to gender-specific reporting and longevity differences. Altogether, the findings demonstrate a demographic and geographic pattern in the epidemiology of disability across Saudi Arabia, providing context for the labour-market, service-access, and social-structural patterns outlined earlier.

**Table 8.** Baseline Population and Disability Prevalence by Age Group, Sex, and Region (Saudi Population 2017) Using the Disability Survey 2017, General Authority for Statistics.

Category	Total Population (n)	With Disability (n)	Total Prevalence (%)	Female Prevalence (%)	Male Prevalence (%)
Age group (years)					
0-4	2 171 164	26 520	1.2	1.3	1.2
5-9	2 121 775	47 087	2.2	1.9	2.3
10-14	1 899 522	95 885	5.0	2.8	2.7
15-19	1 789 169	84 543	4.7	3.0	4.7
20-24	2 018 057	103 903	5.2	3.5	5.3
25-29	1 936 400	65 613	3.4	2.3	3.6
30-34	1 747 732	83 890	4.8	3.5	4.9
35-39	1 527 519	73 475	4.8	3.3	4.9
40-44	1 284 333	66 600	5.2	4.0	5.3
45-49	1 070 154	82 827	7.7	6.9	7.9
50-54	853 081	115 680	13.6	14.5	13.4
55-59	655 841	114 407	17.4	17.3	17.4
60-64	479 334	123 226	25.7	25.6	25.7
65-69	310 359	111 511	35.9	36.0	35.9
70-74	222 818	83 694	37.6	37.8	37.5
75-79	144 079	59 819	41.5	42.0	41.4
80+	177 045	78 779	44.5	44.3	44.6
Region					
Riyadh	4 658 322	363 351	7.8	7.5	8.0
Makkah	4 516 577	336 705	7.5	7.9	7.3
Madinah	1 376 244	98 905	7.2	6.7	7.4
Qassim	1 099 543	65 021	5.9	6.3	5.8
Eastern Region	3 140 362	177 064	5.6	5.2	5.7
Asir	1 750 131	164 441	9.4	9.1	9.5
Tabuk	722 664	43 943	6.1	5.8	6.2
Hail	538 099	43 438	8.1	7.8	8.2
Northern Borders	288 991	12 893	4.5	4.7	4.4
Jazan	1 207 269	87 348	7.2	6.9	7.3
Najran	438 041	12 618	2.9	2.6	3.0
Al-Baha	382 438	25 136	6.6	6.5	6.6
Al-Jouf	379 751	18 549	4.9	5.0	4.8
Total	20 408 362	1 445 723	7.1	6.9	7.3

*Note.* Prevalence (%) = population with disability ÷ total population × 100. Totals refer to Saudi nationals. Percentages rounded to 1 decimal place. The total population excludes 12 143 974 non-Saudis without collected data, including disability information. In all tables and figures, the first 3 age groups—irrelevant for employment rights—are also excluded, so the analysed total is 1 312 261 instead of 1 445 723.

The logistic regression model identifies both structural and individual determinants of employment inclusion among Saudis with disabilities (Table 9). Sex emerges as the strongest predictor: men are more than twice as likely to be employed as women, confirming the gendered division observed in the descriptive tables. Severity and multiplicity of disability substantially reduce the probability of employment, highlighting the functional barriers to labour participation. Access to MLSD services slightly increases employment odds, suggesting a positive but modest institutional inclusion effect. Marital status also contributes positively, implying that social stability may facilitate labour engagement. Age shows a negative gradient, as employment opportunities decline progressively with advancing years. Regional patterns are mild but consistent with baseline data, showing somewhat lower inclusion in southern and peripheral areas such as Asir and Hail. Collectively, these results demonstrate that employment inclusion in Saudi Arabia is shaped by both personal characteristics and contextual inequalities, with gender and severity remaining the dominant structural determinants.

**Table 9.** Logistic Regression Results for Employment Inclusion among Saudis with Disabilities.

Predictor	Adjusted odds ratio (AOR)	95% CI	*P*-value	Interpretation
Sex (male vs female)	2.64	2.47-2.82	<.001	Men are about 2.6 times more likely than women to be employed, consistent with the gender gap shown in Tables 3–5.
Severity (per level increase)	0.58	0.55-0.61	<.001	Employment probability decreases as severity increases, with each level of severity reducing odds by about 40%.
Difficulty scope (multiple vs single)	0.63	0.59-0.68	<.001	Individuals with multiple difficulties have significantly lower employment inclusion odds than those with single difficulties.
Marital status (married vs not married)	1.32	1.22-1.43	<.001	Married persons are modestly more likely to be employed, reflecting household stability and selection effects.
MLSD service access (received vs none)	1.21	1.10-1.34	<.001	Receipt of at least one MLSD service is associated with higher odds of employment, indicating potential institutional inclusion effects.
Age group (per 10-year increase)	0.91	0.88-0.94	<.001	Employment likelihood declines slightly with age, consistent with age-related functional limitations.
Region (ref: Riyadh)				
Makkah	0.97	0.88-1.06	.31	Employment inclusion is similar to Riyadh.
Eastern Region	1.08	0.99-1.17	.07	Slightly higher odds than Riyadh but not significant.
Asir	0.84	0.76-0.93	.002	Significantly lower employment odds compared with Riyadh.
Hail	0.79	0.68-0.91	.001	Employment odds are lower, consistent with higher disability prevalence.
Najran	0.86	0.73-1.02	.09	Slightly reduced odds but not statistically significant.

*Note.* Model statistics: n = 1 312 261; Nagelkerke R^2^ = .29; Hosmer–Lemeshow P = .42. Reference groups: Female, mild severity, single difficulty, not married, no MLSD service, Riyadh region.

Regional coefficients showed modest but meaningful differences in employment inclusion. Compared with Riyadh, odds of employment were lower in Asir and Hail (AORs 0.84 and 0.79), while Makkah, the Eastern Region, and Najran were similar to the reference category. Asir and Hail also had relatively high disability prevalence in Table 8, suggesting that regions with higher prevalence and more rural populations may face greater unmet need and fewer formal employment opportunities for persons with disabilities.

**Figure 2.**
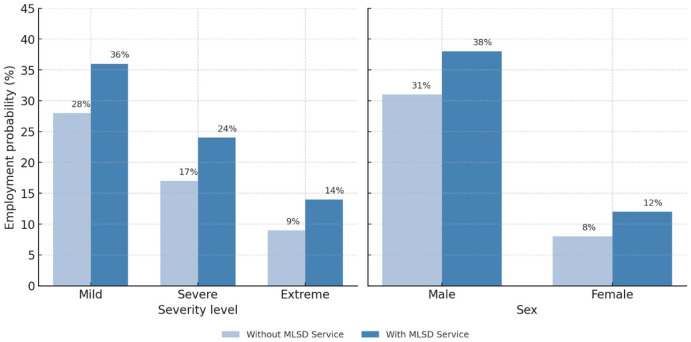
Predicted probabilities of employment among Saudis with disabilities by (left) severity × MLSD service access and (right) sex × MLSD service access (disability survey 2017).

Figure 2 together depict how institutional service access interacts with both functional severity and gender to shape employment outcomes. In the left panel, predicted employment probability decreases with increasing severity but remains consistently higher among individuals receiving MLSD services: from 36% versus 28% for mild difficulties, 24% versus 17% for severe, and 14% versus 9% for extreme. The right panel shows that men have markedly higher employment probabilities than women, yet both groups benefit from MLSD access (38% vs 31% for men, 12% vs 8% for women). The pattern highlights 2 key dynamics—first, that service access positively influences inclusion regardless of severity; and second, that gender remains a dominant structural divide even within the same service framework. These results emphasize the importance of inclusive policy design that not only expands service coverage but also addresses persistent gender and severity inequalities within the Saudi disability employment context.

Scenario simulations indicate that policy levers tied to service access and rehabilitation have measurable but varied impacts on national employment inclusion (Table 10). Expanding MLSD service coverage alone would yield small incremental gains (≈1-3 points), while targeted rehabilitation reducing the proportion of severe cases could increase overall inclusion by ≈4 points. Gender-focussed incentives contribute additional though smaller improvements, highlighting structural barriers that extend beyond service access. A combined strategy integrating MLSD expansion, rehabilitation, and women’s employment initiatives produces the largest estimated employment gains (nearly 5 percentage points). These projections suggest that the most effective pathway towards inclusive employment involves coordinated institutional and social-policy interventions rather than single-channel measures.

**Table 10.** Predicted Employment Gains Under Policy Scenarios Among Saudis with Disabilities.

Policy scenario	Simulation basis	Predicted employment rate (%)	Change from baseline (percentage points)	Interpretation
Baseline (current)	Observed data from 2017	18.5	—	Current national employment inclusion rate among persons with disabilities.
+10% increase in MLSD service coverage	Apply AOR = 1.21 from Table 8	20.0	+1.5	Expanding MLSD services by 10% raises expected employment by about 1.5 points, showing modest institutional impact.
+20% increase in MLSD service coverage	Same AOR = 1.21 (non-linear effect)	21.4	+2.9	Doubling service expansion amplifies gains, indicating moderate scalability of inclusion benefits.
+10% female participation incentive	Using Sex AOR = 2.64 (M vs F) +10% female boost	19.8	+1.3	Female-targeted programs narrow the gender gap slightly but require further support measures.
+10% reduction in severe/extreme cases (via rehabilitation)	Using severity AOR = 0.58	22.7	+4.2	Rehabilitation or early-intervention programs that reduce functional severity yield the largest gains.
Combined policy (+10% services + +10% female incentives)	Joint simulation	23.3	+4.8	Integrated institutional and gender-responsive policies show additive effects on inclusion.

*Note*. Sensitivity analysis was conducted by varying the adjusted odds ratios (AORs) used in the counterfactual simulations by ±10%. For each policy scenario, the baseline probability of employment (*P* = .185) was re-estimated using the same marginal-effects formula applied in the main analysis:

$$\hat{P}=\frac{\mathrm{AOR}\times P}{1-P+(\mathrm{AOR}\times P)}.$$

where *P* is the baseline probability of employment. This approach follows standard marginal-effects simulation methods used in policy analysis.^47,48^

A sensitivity analysis tested whether the counterfactual predictions were robust to moderate uncertainty in the underlying policy effects. When the AOR for MLSD service access (1.21) was varied by ±10%, the predicted employment rate for the +10% service-coverage scenario shifted only slightly, ranging from 18.9% (AOR = 1.089) to 21.1% (AOR = 1.331) compared with the main estimate of 20.0%. For the severity-reduction scenario, predicted employment ranged from 21.8% to 23.7% around the main value of 22.7%. The female-participation scenario was highly stable, varying only between 19.7% and 19.9%. These small deviations indicate that the simulated policy gains are not overly sensitive to moderate variation in the underlying effect sizes.

The AORs for MLSD service access and severity were multiplied by 0.90 and 1.10 to create lower and upper bounds, respectively, and these adjusted values were substituted into the equation while holding all other quantities constant. For the female-participation scenario, the incremental effect was scaled by ±10% using the same proportional adjustment. This approach provides a simple robustness check illustrating how predicted employment rates respond to moderate uncertainty in the underlying effect sizes.

## Discussion

### Key Results

The analyses confirmed the study’s hypotheses and demonstrated clear patterns of inequality in employment inclusion among Saudis with disabilities. First, employment participation varied significantly by sex and severity, with males being more than twice as likely as females to be employed (AOR = 2.64, *P* < .001) and each incremental increase in severity reducing employment odds by ≈40%. Second, access to Ministry of Labor and Social Development (MLSD) services modestly increased the likelihood of employment (AOR = 1.21), indicating limited but positive institutional inclusion. Third, marital status contributed to inclusion differences (AOR = 1.32), suggesting that household stability is associated with higher participation in the labour market. Fourth, simulation models projected tangible employment gains under integrated policy scenarios: expanding MLSD service coverage and female participation incentives could together raise national inclusion by nearly 5 percentage points. Collectively, these findings support the conceptual framework linking labour-market, policy-support, social-structural, and demographic dimensions, showing that gender and functional severity remain the most powerful structural determinants of employment inclusion in Saudi Arabia.

### Interpretation

The study documents a clear employment gap by sex and a steep gradient by severity of functional difficulty. In our data, men had markedly higher odds of employment than women, and employment probability fell steadily from mild to extreme difficulty. These patterns track closely with the Convention on the Rights of Persons with Disabilities’ human-rights framing: equal opportunity at work is not achieved by anti-discrimination provisions alone if structural and attitudinal barriers persist.^1,2^ The ICF helps explain the graded relationship we observed. Disability reflects the interaction of body functions with environments, so when environments are not supportive—transport systems, job design, supervisory practices, or assistive technologies—participation costs rise fastest for those with greater functional limitations.^3-5^ The prevalence and severity patterns we reported therefore point to modifiable factors rather than immutable traits; they suggest that better alignment between work settings and functional diversity could flatten the severity gradient.

The positive, yet modest, association between access to MLSD services and employment is informative for policy. It indicates that service receipt correlates with higher inclusion, but the estimated effect is small compared with the influence of gender and severity. This is consistent with evidence that single-program solutions rarely shift participation unless they operate alongside changes in the physical and social fabric of workplaces—reasonable job accommodations, flexible hours, transport access, and supportive supervision.^4,7^ In the Saudi context, the fit between our findings and the direction of Vision 2030 is noteworthy. Reforms that prohibit workplace discrimination, widen childcare and mobility options, and encourage flexible employment are designed to reduce the non-wage costs of labour-force participation, especially for women.^12,14,17^ The marital-status association we found likely captures some of these household constraints and supports—caregiving responsibilities, income pooling, and social expectations—showing how family structure can mediate access to paid work. Vision 2030s emphasis on women’s economic roles and on enabling infrastructures aligns with this interpretation.

Further, the association between marital status and employment inclusion may reflect selection effects as well as household supports. Individuals with stable employment are more likely to marry or remain married, which may contribute to the pattern observed in the model. Because the data were cross-sectional, the direction of influence could not be established, and the relationship required cautious interpretation.

The scenario exercises strengthen the policy message. Gains were projected when services were expanded and when women’s participation increased, consistent with SDG priorities that link disability inclusion to poverty reduction, decent work, and reduced inequalities.^2,9^ Although simulations are illustrative rather than causal, they translate regression evidence into quantities that matter for planning and budgeting. In practical terms, our results support a twin-track approach: disability-specific supports (benefits, assistive devices, targeted services) combined with mainstream labour reforms that improve job access and retention for women and other under-represented groups. Such an approach fits the ICF’s emphasis on modifying environments and is consistent with national ambitions to diversify the economy while expanding human capital.^15,20^ Finally, the descriptive patterns by age and region in the paper contribute to the epidemiological base that international guidance calls for, helping situate program coverage and barriers in a measurable way for future monitoring.^5,10,11^ It should be noted that the simulations were illustrative rather than predictive, and real-world implementation may involve nonlinearities and behavioural responses that could modify the estimated gains.

Most national inclusion measures were implemented after the 2017 survey year and could not be examined using the data analysed in this study. These reforms included the establishment of the Authority of People with Disability in 2018,^21^ the amendment to Article 3 of the Labour Law prohibiting discrimination in employment,^22^ the *National Policy for Promoting Equal Opportunities and Equal Treatment in Employment and Profession* approved in 2023,^23^ and the *Law on the Rights of Persons with Disabilities* enacted in 2023.^24^ Recent analyses also discussed the role of the 2023 law in advancing disability rights and aligning national policy with international standards.^25^ Because these measures were implemented after the survey year, their effects could not be evaluated using the 2017 dataset.

Publicly released 2023 disability indicators showed clear gender differences in work status among individuals with disabilities.^44^ Only 4.7% of women with disabilities were paid employees compared with 21.3% of men, while 36.2% of women with disabilities were classified as unable to work compared with 20.5% of men. These figures suggested a modest rise in participation among women with disabilities relative to 2017 but confirmed a persistent gender gap. Recent labour-market indicators for the general population provided useful context, with female labour-force participation reaching 33.4% in Q2 2024 and 33.6% in Q3 2024, and the ratio of employed Saudi women to the working-age population remaining near 30%.^45^ Because the 2023 disability release included only percentage summaries and the 2024 indicators were not disability specific, these trends could not be modelled, but they illustrated the broader employment environment surrounding the 2017 disability data.

The regional disparities observed, particularly the lower inclusion in Asir and Hail, were consistent with broader patterns of urbanization, economic diversification, and service infrastructure in Saudi Arabia. Regions with larger rural populations and more limited institutional and transport services are likely to offer fewer accessible jobs and weaker support for persons with disabilities. These findings suggest that national disability employment strategies should include regionally tailored interventions that strengthen service coverage and employer engagement in high-prevalence areas.

### Gender Interaction Considerations

Although this study reports independent effects of sex, severity, and MLSD service access on employment inclusion, the available dataset did not support formal statistical estimation of interaction terms. Nevertheless, evidence from international frameworks and empirical studies suggests that gender may shape how disability severity and service access influence labour-force participation. Prior research shows that workplace norms, social expectations, and caregiving roles intersect with disability status to influence employment opportunities differently for men and women.^2,9,10^ Vision 2030s emphasis on women’s participation further underscores the policy relevance of such intersectional effects. While the present findings indicate that both severity and access to services affect employment outcomes, the extent to which these relationships vary by gender cannot be isolated in grouped data. Future studies using individual-level microdata are needed to evaluate gender-specific mechanisms and to strengthen interpretation of inclusion patterns in the Saudi context.

### Generalizability

Because the analysis draws on a large, nationally representative household survey with weighting by age, sex, and region, the findings are generalizable to Saudi nationals aged ≥ 15 years with functional difficulties in 2017. That said, external validity is bounded by context: non-Saudis were not included; disability was operationalized with national classifications rather than the WHO Model Disability Survey, which can limit cross-country comparability; and the data predate more recent Vision-2030 labour reforms. In line with best practice on external validity and transportability, results should be applied to other settings only where the target population and institutional environment are similar, or after formal assessment of differences between the study sample and the intended target population.^47,48^ Future replications using updated data and, where feasible, harmonized instruments such as the WHO Model Disability Survey would strengthen comparability and policy relevance across countries.^48^

### Limitations

The study’s strengths include its national representativeness, large sample size, and integration of sociodemographic and policy dimensions. These features address a critical gap in national disability research and provide a foundation for future longitudinal and mixed-methods investigations to test causal mechanisms and evaluate ongoing reforms. However, this study has several limitations that should be considered when interpreting its findings.

First, the analysis is based on cross-sectional data, which precludes inference of causality or temporal relationships between disability, service access, and employment outcomes. Cross-sectional designs are widely used in population-based epidemiology for estimating prevalence and associations, yet they cannot capture change or directionality over time.^48^ Second, the Disability Survey 2017 relied on self-reported measures of functional difficulty and service use. Such data are susceptible to recall bias, social-desirability effects, and underreporting—especially for non-visible or stigmatized disabilities—which may result in conservative prevalence estimates.^48^ Third, the dataset did not include several variables that likely affect employment inclusion, such as educational attainment, workplace accommodations, or employer attitudes, leaving room for residual confounding.

Fourth, the policy-simulation component was based on modelled associations rather than experimental data; its projections should therefore be viewed as illustrative scenarios. Moreover, the data reflect the situation in 2017, before the expansion of major Vision 2030 reforms and disability-specific labour programs, so results represent a baseline rather than current conditions. Although disability indicators for 2023 were released, only percentage summaries were available. This limitation prevented the replication of the analytical models with more recent data and restricted the ability to assess the impact of recent policy measures.

A final limitation is that the counterfactual scenarios in Table 10 rely on simplifying assumptions, including constant AORs across policy environments, absence of labour-market saturation, and no spillover effects between policy domains. To assess the robustness of these assumptions, a ±10% sensitivity analysis was applied to the key policy AORs. Predicted employment rates changed modestly across all scenarios—for example, the +10% MLSD-service scenario varied between 18.9% and 21.1%, and the severity-reduction scenario ranged between 21.8% and 23.7%. These modest shifts suggest that the overall direction and magnitude of policy impacts remain stable even under plausible uncertainty, strengthening confidence in the comparative insights drawn from the simulations.

## Conclusion

This study provides a national profile of employment inclusion among Saudis with disabilities, showing that gender, severity, and difficulty scope remain the strongest determinants of participation. Employment is markedly lower among women and among individuals with multiple or severe difficulties, and regional variation reflects broader differences in socioeconomic context. Access to MLSD services is associated with slightly higher inclusion, although the effect is modest and reflects the predominantly custodial nature of available services. The results offer a baseline against which ongoing reforms can be assessed and highlight the need for policies that address both structural barriers and functional limitations. Strengthening employment-oriented supports, improving regional service capacity, and ensuring equitable opportunities for women are central to advancing inclusion.

## Supplemental Material

sj-doc-1-inq-10.1177_00469580261441064 – Supplemental material for Disability and Employment Inclusion in a Transforming Economy: Epidemiological Evidence from National Survey Data Supplemental material, sj-doc-1-inq-10.1177_00469580261441064 for Disability and Employment Inclusion in a Transforming Economy: Epidemiological Evidence from National Survey Data by Abdullah Alduais and Ahmed Alduais in INQUIRY: The Journal of Health Care Organization, Provision, and Financing

sj-xlsx-2-inq-10.1177_00469580261441064 – Supplemental material for Disability and Employment Inclusion in a Transforming Economy: Epidemiological Evidence from National Survey Data Supplemental material, sj-xlsx-2-inq-10.1177_00469580261441064 for Disability and Employment Inclusion in a Transforming Economy: Epidemiological Evidence from National Survey Data by Abdullah Alduais and Ahmed Alduais in INQUIRY: The Journal of Health Care Organization, Provision, and Financing
